# High Prevalence of *bla*_NDM-1_, *bla*_VIM_, *qacE*, and *qacEΔ1* Genes and Their Association with Decreased Susceptibility to Antibiotics and Common Hospital Biocides in Clinical Isolates of *Acinetobacter baumannii*

**DOI:** 10.3390/microorganisms5020018

**Published:** 2017-04-12

**Authors:** Fatma Alzahraa M. Gomaa, Zeinab H. Helal, Mazhar I. Khan

**Affiliations:** 1 Microbiology and Immunology Department, Faculty of Pharmacy, Alazhar University, Cairo 11765, Egypt; fatema5675@hotmail.com; 2 Pathobiology and Veterinary Science Department, University of Connecticut, Storrs, CT 06269-3089, USA

**Keywords:** *Acinetobacter baumannii*, MBL encoding genes, qac genes and biocides resistance

## Abstract

The objective of this study was to evaluate the susceptibility of metallo-β-lactamase (MBL)-producing *Acinetobacter baumannii* (*A. baumannii*) clinical isolates to biocides. We also determined the prevalence and correlation of efflux pump genes, class 1 integron and MBL encoding genes. In addition, *bla*_VIM_, *bla*_NDM-1_, *qacE* and *qacEΔ*1 nucleotide sequence analysis was performed and compared to sequences retrieved from GenBank at the National Center for Biotechnology Information database. *A. baumannii* had a resistance rate to carbapenem of 71.4% and 39.3% and was found to be a MBL producer. The minimum inhibitory concentrations (MICs) of chlorhexidine and cetrimide were higher than the recommended concentrations for disinfection in 54.5% and 77.3% of MBL-positive isolates respectively and their MICs were significantly higher among qac gene-positive isolates. Coexistence of qac genes was detected in 68.1% and 50% of the isolates with *bla*_VIM_ and *bla*_NDM-1_ respectively. There was a significant correlation between the presence of qac genes and MBL-encoding *bla*_VIM_ and *bla*_NDM-1_ genes. Each of the *bla*_NDM-1_, *bla*_VIM_, *qacE* and *qacEΔ*1 DNA sequences showed homology with each other and with similar sequences reported from other countries. The high incidence of Verona integron-encoded metallo-β-lactamases (VIM) and New-Delhi-metallo-β-lactamase (NDM) and qac genes in *A.* *baumannii* highlights emerging therapeutic challenges for being readily transferable between clinically relevant bacteria. In addition reduced susceptibility to chlorhexidine and cetrimide and the potential for cross resistance to some antibiotics necessitates the urgent need for healthcare facilities to periodically evaluate biocides efficacy, to address the issue of antiseptic resistance and to initiate a “biocidal stewardship”.

## 1. Introduction

Recently, *Acinetobacter baumannii* (*A. baumannii*) has received a lot attention due to its increasing prevalence in hospital environments and its high antimicrobial resistance pattern, including resistance to carbapenems. Moreover, it has been identified as a “Red Alert” to hospitalized immunocompromised patients [1]. Resistance to carbapenems is mainly mediated by carbapenem-hydrolyzing β-lactamases. A variety of β-lactamases which include metallo-β-lactamases (MBL), have emerged as the most worrisome mechanism of resistance among Gram-negative bacteria, which pose a therapeutic challenge to health care settings [2,3,4]. MBL encoding genes, *bla*_IMP_, *bla*_VIM_, *bla*_GIM_, *bla*_SIM_, and *bla*_KMH_ and *bla*_NDM-1_, are harbored by class 1 integrons and are found as gene cassettes [5,6]. The VIM-type β-lactamase (Verona integron-encoded metallo-β-lactamases) *Acinetobacter* spp. was initially described in Europe [7,8] and has been reported worldwide. New-Delhi-metallo-β-lactamase (NDM) is a recently discovered transferable molecular class B β-lactamase, first identified in New Delhi, India [9]. The emergence and dissemination of NDM-producing isolates have been reported in several countries, including USA, Canada, Sweden, UK, Austria, Belgium, France, the Netherlands, Germany, Japan, Australia, Africa and some Middle Eastern countries [3,10]. The *bla*_NDM-1_ gene has been detected on different large plasmids, which were readily transferable among bacteria [6]. Most *bla*_NDM-1_-positive bacteria are resistant to almost all antibiotics and carry different supplementary mechanisms of resistance [11,12], making *bla*_NDM-1_-producing bacteria a significant clinical and public health threat. As a result, reliable detection and surveillance of NDM-producing bacteria is crucial.

Moreover, the co-resistance to antibiotics and biocides might contribute to the epidemic prevalence of resistant strains within healthcare settings [13]. Reduced susceptibility to these antimicrobials has been reported in various Gram-negative bacteria [14,15,16]. 

One of the biocides resistance mechanisms is the expression of efflux systems involving qac genes (*qacE* and *qacEΔ*1). *A. baumannii* resembling other Gram-negative bacteria harbors multidrug transporter efflux systems. Qac genes are widely propagated in Gram-negative bacteria [17,18] basically due to high spread of plasmid-mediated class 1 integrons, which commonly include *qacEΔ*1 [17]. Qac genes have been frequently noticed in combination with genes coding for resistance to β-lactams (including carbapenemases), aminoglycosides, trimethoprim, sulphonamides, and chloramphenicol [19,20,21,22,23]. The ability of qac genes to offer resistance to antibiotics remains unspecified. Nevertheless, a close association between resistance to biocides and antibiotics can be explained by the fact that the class 1 integrons (mobile genetic elements) hosts a variety of antibiotic resistance genes [23,24]. Owing to these facts, there is a rational concern that the inadequate use of biocides could select for antibiotic-resistant bacteria in Gram-negative bacteria [25,26].

Although many reports focus on the increasing resistance of *A. baumannii* strains to antibiotics, few studies have investigated the susceptibility of *A. baumannii* to biocides [27,28,29]; also, there is little information available that weighs the risks of antibiotic resistance induced by increased resistance to biocides in Egypt. Undoubtedly, understanding *A. baumannii* susceptibility to disinfectants and its correlation with antibiotic resistance will contribute to the control of this microbe in hospitals.

We aimed to investigate the susceptibility of MBL *A. baumannii* producer, as one of the troublesome resistant clinical isolates, to biocides. In addition, we evaluate the correlation between carriage rates of qac genes and the prevalence of MBL encoding genes and class 1 integrons.

## 2. Materials and Methods

### 2.1. Bacterial Isolates

A total of 56 non-consecutive *A. baumannii* clinical isolates were included in the present study. The clinical isolates were recovered from different specimens (including blood, pus/wound swabs, throat swabs, chest tube, nasal swabs, sputum and urine) submitted to the Clinical Pathology Department, National Cancer Institute, Cairo, Egypt, for routine culture. The specimens were collected from immunocompromised patients over a period of 9 months from June 2014 to March 2015. Only cases confirmed to be hospital acquired infections were included. Phenotypic identification of isolates was performed using VITEK automated system and the isolates were identified genotypically through detection of the *bla*_OXA-51_-like gene by PCR. 

### 2.2. Antimicrobial Susceptibility Testing

Antibiotic sensitivity to 17 antibiotics belonging to five different classes was tested by modified Kirby-Bauer disc diffusion method. The results were interpreted according to Clinical and Laboratory Standards Institute guidelines [30]. An intermediate susceptibility was considered as resistant. The antibiotic discs (Oxoid, UK) used in this study were amoxicillin/clavulanic acid (AMC)—20/10 mcg, amikacin (AK)—30 mcg, cefazoline (KZ)—30 mcg, ceftazidime (CAZ)—30 mcg, cefotaxime (CTX)—30 mcg, cefotetan (CTT)—30 mcg, gatifloxacin (GAT)—5 mcg, tobramycin (TOB)—10 mcg, cefuroxime (CXM)—30 mcg, levofloxacin (LEV)—5 mcg, ceftriaxone (CRO)—30 mcg, meropenem (MEM)—10 mcg, nitrofurantoin (F)—30 mcg, imipenem (IMP)—10 mcg, cefepime (FEP)—30 mcg and trimethoprim/sulfamethoxazole (SXT)—1.25/23.75 mcg, ticarcillin/Clavulinic (TIM)—75/10 mcg, colistin (CL)—10 mcg and tigecycline (TGC)—15 mcg. *Escherichia coli* ATCC 25922 was used as the reference strain.

### 2.3. Phenotypic Detection of MBL

E-test MBL strips (AB Biodisk, Solna, Sweden) were used in accordance with the manufacturer’s instructions to investigate MBL production. MIC ratio of imipenem to imipenem/EDTA of 8 or the presence of a phantom zone was taken as a positive result. 

### 2.4. Determination of Disinfectants/Antiseptics Susceptibility by the Broth Macrodilution Method

The biocides selected for testing were the chemical disinfectants/antiseptics recommended for patient-care items and instruments (most widely used biocides). Chlorhexidine (0.3%, CLX), cetrimide (3%), and aqueous povidone-iodine (10% *w*/*v*, PVP-I_2_) solution were included. The MICs of the disinfectants/antiseptics were determined by the broth macrodilution method according to [31]. Each disinfectant solution was diluted by a serial two-fold dilution method using Muller Hinton broth (MHB) (Table 1). The positive control tube contained 0.1 mL inoculum in 1 mL MHB and should show evidence of bacterial growth (turbidity). The negative control tube contained MHB and the disinfectant to be tested and should not show bacterial growth (clear).

### 2.5. PCR and DNA Sequencing

Genomic DNA was extracted from *A. baumannii* isolates using Qiaamp Mini DNA kit (Qiagen, Hilden, Germany). *bla*_OXA-51_-like gene, *Intl*1, *bla*_VIM_, *bla*_NDM-1_, *bla_I_*_MP_, *bla*_GIM_, *bla*_SIM_, *bla*_SPM_, *qacE*, *qacΔE*1 and *cepA* were amplified for *A. baumannii* isolates using the primers listed in Table 2. The primers were prepared by Invitrogen (Grand Island, NY, USA). GoTaq Green Master Mix (Promega, Madison, WI, USA) was used in all PCR assays. The PCR products were separated through a 1.5% agarose gel by electrophoresis. Amplified products were cut and purified using Qiaquick gel purification kit (Qiagen, Hilden, Germany) and sequenced at DNA Biotechnology Facility at University of Connecticut, USA (using the Genetic analyzer 3030X1 (Applied Biosystems, CA, USA) with a big Dye terminator cycle sequencing kit). Sequences in GenBank (http://blast.ncbi.nlm.nih.gov/Blast.cgi) were used to identify and compare the genes detected in this study. The *bla*_VIM_, *bla*_NDM-1_, *qacE* and *qacΔE*1 specified DNA amplicon sequences and similar sequences recovered from NCBI GenBank database were used to create the phylogenetic trees in order to understand the genetic relatedness of the study sequences. Alignment of these genes nucleotide sequence was performed using the DNASTAR program (DNASTAR Inc., Madison, WI, USA).

### 2.6. Statistical Analysis

Statistical analysis was done using SPSS version 20 (IBM^®^, New York, NY, USA). Chi-square test was performed. A *p*-value of <0.05 was considered indicative of a statistically significant difference.

## 3. Results

### 3.1. Characteristics of Isolates and Their Antibiotics Susceptibility

Out of 56 *A. baumannii* isolates, 22 were recovered from blood (39.3%), 18 from pus/wound swabs (32.1%), nine from sputum (25%), three from a chest tube (5.4%), two from throat swabs (3.6%), one from a urine specimen (1.8%) and one from a nasal swab (1.8%). Table 3 shows the antimicrobial suscebtipility patterns of 56 *A. baumannii* isolates. *A*. *baumannii* resistance rate to carbapenem (imipenem and meropenem) was 71.4% (40/56) and 39.3% (22/56) were MBL producers. Forty-nine isolates were MDR (87.5%) and seven were extensively drug resistant (XDR) (12.5%). “MDR *Acinetobacter* spp.” is defined as isolate non-susceptible to at least one agent in three or more antimicrobial categories. “XDR *Acinetobacter* spp.” is defined as an *Acinetobacter* spp. isolate that is resistant to all standard antimicrobial agents except colistin or tigecycline [38]. Colistin and tigecycline were totally effective against all tested isolates. All MBL-producing isolates were MDR and 31.8% of them were XDR. Antibiotic susceptibility results for MBL-producing *A. baumannii* are shown in Table 4. 

### 3.2. Detection of MBL-Encoding Genes

**Figure 1:**
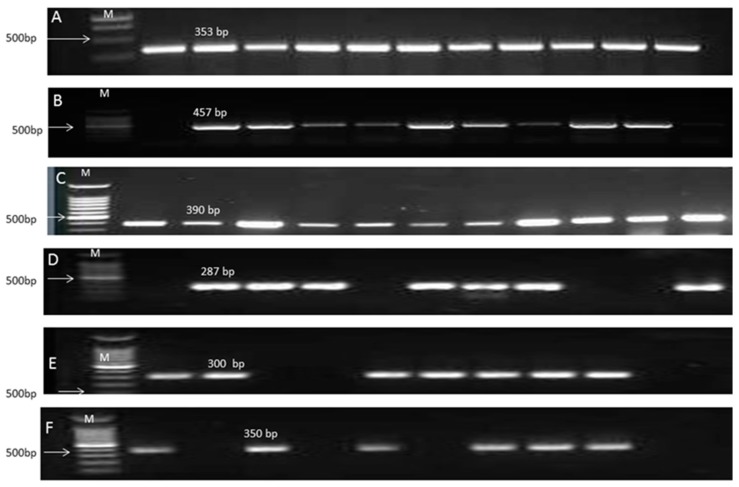
Gel electrophoresis of the PCR amplified products of *bla*_Oxa51_-like gene, *intl*1, MBL genes and Qac genes of *A. baumannii* isolates. (**A**) PCR amplified products of *bla*_Oxa51_-like gene with 353 bp amplification fragment. M: 1 Kbp DNA ladder; (**B**) PCR amplified products of *Intl*1 gene with 475 bp amplification fragment. M: 100 bp DNA ladder; (**C**) PCR amplified products of *bal*_VIM_ gene with 390 bp amplification fragment. M: 100 bp DNA ladder; (**D**) PCR amplified products of *bla*_NDM-1_ gene with 287 bp amplification fragment. M: 100 bp DNA ladder; (**E**) PCR amplified products of *qacE* gene with 350 bp amplification fragment. M: 100 bp DNA ladder; (**F**) PCR amplified products of *qacEΔ*1 gene with 300 bp amplification fragment. M: 100 bp DNA ladder.

The amplified products of *bla*_OXA-51_-like gene, *Intl*1 gene and MBL-positive genes for the *A. baumannii* isolate were shown in Figure 1. The intrinsic β-lactamase gene, *bla*_OXA-51_-like, was amplified from all *A. baumannii* isolates. Of the positive MBL isolates, MBL-encoding gene *bla*_VIM_ was identified in 86.4% (19/22) of MBL-producing isolates and *bla*_NDM-1_ was identified in 13 (59.1%) isolates. Coexistence of *bla*_VIM_ and *bla*_NDM-1_ was encountered in 13 isolates (all *bla*_NDM-1_-positive isolates). In this study, MBL *bla*_IMP_, *bla*_GIM_, *bla*_SIM_, and *bla*_SPM_ were not detected. All the MBL-producing isolates were positive for class 1 integron.

### 3.3. Correlation of Efflux Pump Genes with MIC of Tested Biocides

In the present study, MBL *A. baumannii* isolates were effectively inhibited by the user’s defined concentrations of PVP-I_2_, whereas the MICs of 54.5% and 77.3% of the tested isolates for CLX and cetrimide, respectively, were higher than the actual concentrations recommended for disinfection. The amplified products of qac genes were shown in Figure 1E,F.

Among MBL-positive isolates, *qacE* and *qacΔE*1 efflux genes were present in 10 (45.5%) and 15 (68%) isolates respectively, whereas *cepA* gene was not detected. In general, for qac-positive isolates, the MICs ranged from 47 to 750 μg/mL and 470 to 7500 μg/mL for CLX and cetrimide, respectively. For qac-negative isolates, MIC ranged from 23.4 to 94μg/mL and 470 to 1875 μg/mL for CLX and cetrimide, respectively. The MICs of PVP-I_2_ ranged from 390 to 12,500 μg/mL with both qac-positive and negative isolates (Table 4). There was a significant correlation between qac genes carriage and increased MICs of CLX and cetrimid (*p* = 0.0096 and 0.0085, respectively) as showed in Table 5.

### 3.4. Correlation of bla_VIM_ and bla_NDM-1_ Genes with qac Genes

As indicated in Table 6, coexistence of *bla*_VIM_ and qac genes was detected in 15 isolates, while *bla*_NDM-1_ was concomitant in 11 isolated with qac genes, and coexistence of the *qacE* and *qacEΔ*1 genes was encountered in 10 of the MBL-positive isolates. There is significant correlation between qac and the carriage of both *bla*_VIM_ and *bla*_NDM-1_ genes (*p* = ‎0.0064 and ‎0.0467, respectively‎). 

### 3.5. Nucleotides Sequence and Phylogenetic Analysis

Sequencing of intrinsic *bla*_VIM_ and *bla*_NDM-1_ confirmed that the nucleotide sequences obtained were identical to genes for VIM and NDM for *A. baumannii*. In the same way, the nucleotide sequences of class І integrin, qacE and qacEΔ1 also agreed with the results expected by the PCR analysis.

**Figure 2:**
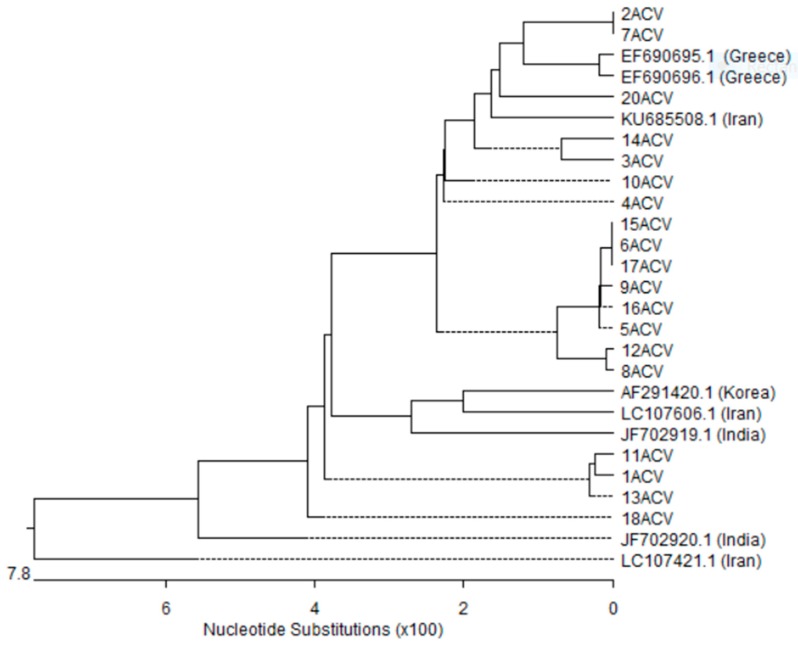
Phylogenetic analysis based on *bla*_VIM_ gene sequences obtained from the nineteen *A. baumannii* isolates in this study and eight sequences retrieved from GenBank database. Phylogenetic tree was created with DNASTAR MegAlign by ClustalW (weighted) method. ACV; *bla*_VIM_ gene isolated in this study and the other sequences were taken from Genbank.

The nucleotide sequences of the *bla*_VIM_ gene-positive isolates were highly similar, with sequence identities of 90.8–100%. The genetic divergence and homogeneity of the *bla*_VIM_ sequences are apparent in the phylogenetic tree Figure 2. Among the 19 *bla*_VIM_ gene sequences, there were nine different clusters. Those from isolates number 14, 18 and 20 were found to form distinct clusters, while six clusters formed from the rest of isolates’ shared similarity.

*bla*_VIM_ sequences were found to have genetic relationship with sequences from other countries; one from Korea (GenBank accession number: AF291420.1), two from Greece (GenBank accession numbers: EF690695.1 and EF690596.1), two from India (GenBank accession numbers: JF702919.1 and JF702920.1) and three from Iran (GenBank accession numbers: KU6855081.1, LC107606.1 and LC107421.1).

**Figure 3:**
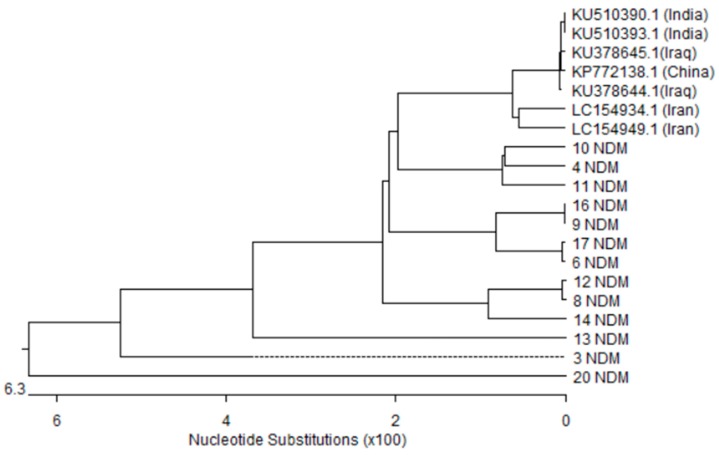
Phylogenetic analysis based on *bla*_NDM-1_ gene sequences obtained from the thirteen *A. baumannii* isolates in this study and seven sequences retrieved from GenBank database. Phylogenetic tree was created with DNASTAR MegAlign by ClustalW (weighted) method. NDM; *bla*_NDM_ gene isolated in this study and the other sequences were taken from Genbank.

The nucleotide sequences of the *bla*_NDM-1_ gene in 13 out of 22 *A. baumannii* isolates were highly similar, with sequence identities of 88–100%. *bla*_NDM-1_ sequences of isolates number 3, 13 and 20 were found to form distinct clusters whilst the other isolates shared similarity in four diverse clusters. These sequences were closely related to *bla*_NDM-1_ sequences from other countries: one from China (GenBank accession number: KP772138), two from Iran (GenBank accession numbers: LC154934.1 and LC154949.1) and two from India (GenBank accession numbers: KU510390 and KU510393). In addition, two sequences reported from Iraq (GenBank accession numbers: KU378644.1 and KU378645.1) were also clearly apparent in the tree (Figure 3). 

**Figure 4:**
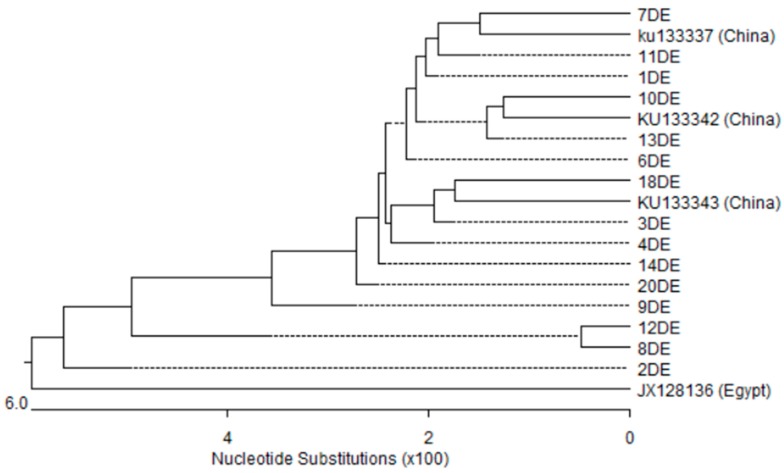
Phylogenetic analysis based on *qacΔE*1 gene sequences obtained from the fifteen *A. baumannii* isolates in this study and four sequences retrieved from GenBank database. Phylogenetic tree was created with DNASTAR MegAlign by ClustalW (weighted) method. DE; *qacEΔ*1 gene isolated in this study and the other sequences were taken from Genbank.

**Figure 5:**
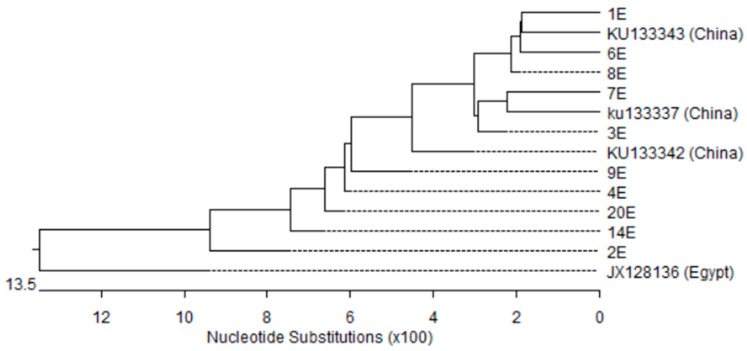
Phylogenetic analysis based on *qacE* gene sequences obtained from the ten *A. baumannii* isolates in this study and four sequences retrieved from GenBank database. Phylogenetic tree was created with DNASTAR MegAlign by ClustalW (weighted) method. E; *qacE* gene isolated in this study and the other sequences were taken from Genbank.

The nucleotide sequences of the *qacEΔ*1 gene (15/22) and *qacE* gene (10/22) among *A. baumannii* isolates were highly similar, with sequence identities of 88.9–100% and 88.2–100%, respectively. Phylogenetic analysis based on *qacE* and *qacEΔ*1 genes (Figure 4 and Figure 5) showed three and five different clusters respectively. Only isolate number 9 formed distinct cluster of both genes. The nucleotide sequences of *qacEΔ*1 and *qacE* shared genetic similarity with sequences from other countries: three from China (GenBank accession numbers: ku133343.1, ku133342.1, ku133337.1) along with one sequence reported from Egypt (GenBank accession number: JX128136.1). 

## 4. Discussion

The worldwide increased occurrence of carbapenem-resistant *A*. *baumannii* infections in healthcare settings has led to a greater alertness of the threat of hospital acquired infections. In the current study, there was high frequency of carbapenem-resistant *A. baumannii* strains that may be attributed to the extensive misuse of carbapenems. Resistance to carbapenem in clinical isolates of *A. baumannii* has been reported in Egypt [39,40,41,42,43]. At this time, there are limited selections of treatment options for carbapenem-resistant *A. baumannii* infection, according to our results; colistin and tigecycline are considered the last choice to control (100% sensitivity). This finding has been also reported in different studies [4,42,43,44,45].

The high frequency of MBL *A. baumannii* detected in the study is not common, since two previous separate studies have shown 0% and 2.5% MBL activity among *A. baumannii* isolated in Egypt [42,43]. Resistance due to MBL production has a potential for rapid dissemination, since it is often plasmid-mediated [40]. The current study showed that all MBL-producing isolates were MDR that exhibited high resistance to beta-lactams, aminoglycosides, and quinolones. 

Because of its ability to spread, carbapenem resistance related to VIM and NDM β-lactamase production has become a serious concern. MBL VIM has been reported intermittently in Egypt [40,42]. The nucleotide sequences of the *bla*_VIM_ gene from the positive isolates were highly similar, with sequence identities of 90.8–100%. *bla*_VIM_ originated from Greece and South Korea [46]. *bla*_VIM_ nucleotide sequences showed high similarity with these two countries and other countries like India and Iran. Horizontal transfer of VIM-encoding genes can be inferred from the presence of the same integron in genetically non-related isolates in different species and genera [47]. The most recent MBL identified in *A. baumannii* are the emerging NDM enzymes. The *bla*_NDM-1_ genes have been reported in *A. baumannii* from India [48] and Germany [49]. Kaase et al. [50] reported the first NDM-producing *A. baumannii* in Egypt from a patient who transferred from Egypt to Germany without obvious link with the Indian subcontinent. After recent identification of NDM-producing isolates in Iraq [51] and the Sultanate of Oman, the clinical case suggests that NDM-producing bacteria disseminated in the Middle East countries [3]. These isolates were resistant to high levels of all β-lactams, including carbapenems. In our study, 68% of the isolates carried *bla*_NDM-1_ gene. The nucleotide sequences of *bla*_NDM-1_ gene showed high similarity between isolates (with sequence identities of 88–100%) and with the sequence of *bla*_NDM-1_ gene identified from other countries (mainly including India, Iran and Iraq). *bla*_VIM_ and *bla*_NDM-1_ genes are horizontally transferable as they are inserted in integrons, and some of these integrons are located on conjugative plasmids [46]. The large number of trips between the countries combined with the ease of resistance transmission among bacteria led us to consider that our isolates may share genetic similarity of *bla*_VIM_ and *bla*_NDM-1_ with other countries. 

The increase in reduced susceptibility to antibiotics is paralleled by a similar trend in reduced susceptibility to biocides [15,52]. Antiseptic resistance has been reported for several agents including quaternary ammonium compounds and biguanides [53]. Interestingly, more than half of the tested isolates showed higher MICs of CLX and cetrimide than the actual concentrations recommended for disinfection. This may be attributed to the extensive use of these types of disinfectants in the routine infection control in Egypt. Moreover, broader and more indiscriminate use of biocides in healthcare facilities could drive the emergence of new genetic elements, with unpredictable consequences for human welfare. 

Resistance to biocides is attributable to production of efflux proteins encoded by plasmid-mediated genes such as qac genes [54]. The MICs of CLX and cetrimid significantly correlated to qac genes carriage. 

We found that the majority of MBL-positive *A. baumannii* isolates harbor at least one of the qac genes tested and *qacEΔ*1 was the most prevalent, with high nucleotide sequence identities between isolates. High levels of *qacE∆*1 carriage have been reported in clinical isolates of Acinetobacter, and it has been suggested that the increase in both antibiotic and antiseptic resistance in this organism is related to the presence of this gene [55]. Resistance to several antibiotics has frequently been reported in different clinical isolates in association with qac genes [56,57]. The acquisition of efflux pumps and co-selection of antibiotic resistance genes through their linkage with biocide resistance determinants on the same mobile element are the mechanisms by which biocide resistance increases the distribution of antibiotic-resistant bacteria [58,59,60,61].

Increased frequency of biocide resistance genes in *A. baumannii* from clinical specimens points to the possibility that the hospital atmosphere could apply selective pressure for presence of these strains. Biocide resistance may allow persistence of organisms in the presence of small levels of biocide and contribute to the survival of MDR strains [62]. Different studies have shown that disinfectant-resistance gene expression can be induced by exposure to subinhibitory concentrations of biocides [53]. Reduction in effectiveness can allow more strains harboring qac genes to survive and spread these genes among *A. baumannii*, which may explain the higher incidence of these resistance genes. 

The genetic similarity between isolates was apparent in the nucleotide sequences of *bla*_VIM_, *bla*_NDM-1_, qacE and qacEΔ1 and suggests a common genetic vehicle related dissemination of the β-lactamase genes and efflux pump genes. Furthermore, these sequences also showed similarities with those previously reported from different geographical areas. The class 1 integrons are the most common mechanism by which bacteria are able to move resistance gene cassettes from one bacterium to another. The importance of horizontal transfer of resistance genes through mobile genetic elements and its relationship with increased incidence of multidrug-resistant *A. baumannii* in hospitals is a critical issue. We evaluated intl1 gene which comprises the genetic platforms of class 1 integrons (genetic mobile elements). We found that all MBL-producing *A. baumannii* isolates harbor the intl1 gene. Since efflux genes are located on mobile genetic elements along with specific antibiotic resistance genes [63], cross-resistance and co-resistance may be acquired together [64]. The MBL genes are mostly harbored by class 1 integrons and the integrons may be targeted as epidemiological molecular markers for identifying and surveying MBL-producing Gram-negative bacilli [2].

In conclusion, a prevalence of MBL-producing *A. baumannii* isolates was observed in this study. The *bla*_VIM_-specific amplicons in these isolates were found to be genetically similar to each other. As well, the genetic similarity between isolates was obvious in the nucleotide sequences of *bla*_NDM-1_ genes. Furthermore, these sequences also showed similarities with those previously reported from other countries. Thus, this study argues for the urgent implementation of strict control measures to prevent the spread of resistance genes and that it warrants the need for constant surveillance. It is crucial to highlight the need for health care facilities to assess the antimicrobial effectiveness of biocides periodically to overcome dissemination of MBL-producing *A. baumannii* with reduced sensitivity to biocides. Reduced susceptibility to CHX and cetrimide and the potential for cross resistance to some antibiotics highlights the need to restrict the use of these biocides. Strains harboring qac genes may be more likely to survive the disinfection process and serve as a source of nosocomial outbreaks. The use of biocides may have driven the fixation and spread of the class 1 integrons, and now may contribute to antibiotic resistance. 

## Figures and Tables

**Table 1:** The chemical agents, their starting concentrations and dilutions used for minimum inhibitory concentration (MIC) test.

Chemical Agents	Recommended Dilution *	Starting Concentration	Dilutions
Chlorhexidine digluconate (CLX)	0.0075%	0.3%	0.15%, 0.075%, 0.0375%, 0.0187%, 0.00937%, 0.00468%, 0.00234%, 0.00172%
Cetrimide	0.075%	3%	1.5%, 0.75%, 0.375%, 0.187%, 0.094%, 0.046%, 0.023%.
Povidone-iodine (PVP-I_2_)	5%	10%	5%, 2.5%, 1.25%, 0.625%, 0.3125%, 0.156%, 0.078% and 0.039%

* According to manufacturer instructions.

**Table 2:** List of primers used in the study.

Gene Name	Sequence 5′-3′	PCR Products	Reference	Annealing Temperature
*qacE*	Forward-CCCGAATTCATGAAAGGCTGGCTT Reverse-TAAGCTTTCACCATGGCGTCGG	350 bp	[14]	55 °C
*qacΔE*1	Forward-TAGCGAGGGCTTTACTAAGC Reverse-ATTCAGAATGCCGAACACCG	300 bp	[32]	55 °C
*cepA*	Forward-CAACTCCTTCGCCTATCCCG Reverse-TCAGGTCAGACCAAACGGCG	1058 bp	[33]	62 °C
*bla*_Oxa51-_*like*	Forward-TAATGCTTTGATCGGCCTTG Reverse-TGGATTGCACTTCATCTTGG	353 bp	[34]	60 °C
*Intl*1	Forward-GCATCCTCGGTTTTCTGG Reverse-GGTGTGGCGGGCTTCGTG	457 bp	[35]	60 °C
*bla*_IMP_	Forward-CTACCGCAGCAGAGTCT TTG Reverse-AACCAGTTTTGCCTTACCAT	587 bp	[36]	55 °C
*bla*_NDM-1_	Forward-GGCGGAATGGCTCATCACGA Reverse-CGCAAC ACAGCCTGACTTTC	287 bp	[6]	58 °C
*bal*_VIM_	Forward-GATGGTGTTTGGTCGCATA Reverse-CGAATGCGCAGCACCAG	390 bp	[37]	52 °C
*bla*_SPM_	Forward-AAAATCTGGGTACGCAAACG Reverse-ACATTATCCGCTGGAACAGG	271 bp		
*bla*_GIM_	Forward-TCGACACACCTTGGTCTGAA Reverse-AACTTCCAACTTTGCCATGC	477 bp		
*bla*_SIM_	Forward-TACAAGGGATTCGGCATCG Reverse-TAATGGCCTGTTCCCATGTG	570 bp		

**Table 3:** Antimicrobial susceptibility patterns of *Acinetobacter baumannii* enrolled in the study.

Antimicrobial Agent	*A. baumannii* (56 Isolates)		
	S No (%)	I No (%)	R No (%)
Amikacin	9 (16.1)	3 (5.3)	44 (78.6)
Amoxicillin/clavulanic acid	0 (0.0)	0 (0.0)	56 (100)
Cefazolin	0 (0.0)	0 (0.0)	56 (100)
Cefepime	4 (7.1)	0 (0.0)	52 (92.9)
Cefotaxime	1 (1.8)	5 (8.9)	50 (89.3)
Cefotetan	1 (1.8)	2 (2.6)	53 (94.6)
Ceftazidime	4 (7.1)	1 (1.8)	51 (91.1)
Ceftriaxone	5 (8.9)	2 (2.6)	49 (87.5)
Cefuroxime	1 (1.8)	1 (1.8)	54 (96.4)
Imipenem	16 (28.6)	3 (5.3)	37 (66.1)
Levofloxacin	13 (23.2)	7 (12.5)	36 (64.3)
Meropenem	16 (28.6)	1 (1.8)	39 (69.6)
Gifloatxacin	8 (14.3)	5 (8.9)	33 (58.9)
Nitrofurantoin	0 (0.0)	0 (0.0)	56 (100)
Ticarcillin/clavulanic acid	6 (10.7)	1 (1.8)	49 (87.5)
Tobramycin	16 (28.6)	6 (10.7)	34 (60.7)
Trimethoprim/sulfamethoxazole	4 (7.1)	0 (0.0)	52 (92.9)
Tigicyclin	56 (100)	0 (0.0)	0 (0.0)
Colistin	56 (100)	0 (0.0)	0 (0.0)

**Table 4:** Antibiotic susceptibility, MICs of biocides and PCR results of metallo-β-lactamase (MBL)-positive *A. baumannii* isolates.

Isolate Number	Type of Specimen	Biocide Resistance Genes			MIC of Biocide μg/mL			MBL Genes		Intl1 Gene	Antimicrobial Susceptibility																				
		*qacE*	*qacEΔ1*	*cepA*	PVP-I2	CLX	Cetrimide	*bla*_NDM_	*bla*_VIM_		Ak	AMC	KZ	FEP	CTX	CTT	CAZ	CRO	CXM	GAT	IMP	MEM	LEV	F	TIM	TOB	SXT	TGC	CL	Category	MBL
1	Throat Swab	+	+	-	‎0.6‎‎25‎	‎0.0‎‎75‎	‎0.18‎‎7‎	-	+	+	R	R	R	R	R	R	R	R	R	I	R	R	I	R	R	R	R	S	S	MDR	+
2	Sputum	+	+	-	‎0.6‎‎25‎	‎0.0‎‎375‎	‎0.75‎	-	+	+	R	R	R	R	R	R	R	R	R	R	R	R	R	R	R	R	R	S	S	XDR	+
3	Throat Swab	+	+	-	‎1.2‎‎5‎	‎0.0‎‎09‎	‎0.04‎‎9‎	+	+	+	R	R	R	R	R	R	R	R	R	R	I	R	R	R	R	R	R	S	S	MDR	+
4	Blood	+	+	-	‎1.2‎‎5‎	‎0.0‎‎046‎	‎0.75‎	+	+	+	I	R	R	R	R	R	R	R	R	R	R	R	S	R	R	R	R	S	S	MDR	+
5	Sputum	-	-	-	‎1.2‎‎5‎	‎0.0‎‎09‎	‎0.09‎‎8‎	-	+	+	R	R	R	R	R	R	R	R	R	I	R	R	I	R	R	S	R	S	S	MDR	+
6	Pus	+	+	-	‎0.6‎‎25‎	‎0.0‎‎375‎	‎0.09‎‎8‎	+	+	+	S	R	R	R	R	R	R	R	R	R	R	R	I	R	R	S	S	S	S	MDR	+
7	Blood	+	+	-	‎0.6‎‎25‎	‎0.0‎‎09‎	‎0.75‎	-	+	+	R	R	R	R	R	R	R	R	R	I	I	R	I	R	R	I	R	S	S	MDR	+
8	Sputum	+	+	-	‎1.2‎‎5‎	‎0.0‎‎046‎	‎0.75‎	+	+	+	R	R	R	R	R	R	R	R	R	R	R	R	R	R	R	R	R	S	S	XDR	+
9	Blood	+	+	-	‎1.2‎‎5‎	‎0.0‎‎09‎	‎0.09‎‎8‎	+	+	+	R	R	R	R	R	R	R	R	R	R	R	R	R	R	R	R	R	S	S	XDR	+
10	Pus	-	+	-	‎0.6‎‎25‎	‎0.0‎‎75‎	‎0.18‎‎7‎	+	+	+	S	R	R	R	R	R	R	R	R	S	R	R	R	R	R	R	R	S	S	MDR	+
11	Pus	-	+	-	‎1.2‎‎5‎	‎0.0‎‎09‎	‎0.75‎	+	+	+	R	R	R	R	R	R	R	R	R	R	R	R	R	R	R	R	R	S	S	XDR	+
12	Blood	-	+	-	‎1.2‎‎5‎	‎0.0‎‎09‎	‎0.18‎‎7‎	+	+	+	R	S	R	R	R	R	R	R	R	S	R	R	S	R	R	R	R	S	S	MDR	+
13	Sputum	-	+	-	‎1.2‎‎5‎	‎0.0‎‎046‎	‎0.09‎‎8‎	+	+	+	R	R	R	R	R	R	R	R	R	R	I	R	R	R	R	R	R	S	S	MDR	+
14	Pus	+	+	-	‎1.2‎‎5‎	‎0.0‎‎09‎	‎0.18‎‎7‎	+	+	+	S	R	R	R	R	R	R	R	R	R	R	R	R	R	R	R	R	S	S	MDR	+
15	Pus	-	-	-	‎1.2‎‎5‎	‎0.0‎‎023‎	‎0.04‎‎9‎	-	+	+	R	R	R	R	R	R	R	R	R	R	R	R	R	R	R	S	R	S	S	MDR	+
16	Blood	-	-	-	‎0.0‎‎39‎	‎0.0‎‎023‎	‎0.18‎‎7‎	+	+	+	R	R	R	R	R	R	R	R	R	R	R	R	R	R	R	R	R	S	S	XDR	+
17	Pus	-	-	-	‎0.6‎‎25‎	‎0.0‎‎046‎	‎0.04‎‎9‎	+	+	+	S	R	R	R	R	R	R	R	R	S	R	R	I	R	R	R	R	S	S	MDR	+
18	Sputum	-	+	-	‎1.2‎‎5‎	‎0.0‎‎046‎	‎0.18‎‎7‎	-	+	+	R	R	R	R	R	R	R	R	R	R	R	R	I	R	R	R	R	S	S	MDR	+
19	Chest Tube	-	-	-	‎0.0‎‎78‎	‎0.0‎‎046‎	‎0.18‎‎7‎	-	-	+	R	S	R	R	R	I	R	R	R	S	R	R	R	R	R	R	R	S	S	MDR	+
20	Chest Tube	+	+	-	‎0.0‎‎39‎	‎0.0‎‎75‎	‎0.75‎	+	+	+	R	R	R	R	R	R	R	R	R	R	R	R	R	R	R	R	R	S	S	XDR	+
21	Pus	-	-	-	‎0.0‎‎78‎	‎0.0‎‎023‎	‎0.04‎‎9‎	-	-	+	R	R	R	R	R	I	R	R	R	R	R	R	I	R	R	R	R	S	S	MDR	+
22	Nasal Swab	-	-	-	‎0.6‎‎25‎	‎0.0‎‎023‎	‎0.04‎‎9‎	-	-	+	S	R	R	R	R	R	R	R	R	R	R	R	R	R	R	R	R	S	S	MDR	+

PVP-I_2_: Povidone-iodine; CLX: Chlorhexidine; AK: Amikacin; AMC: Amoxicillin/clavulanic acid; KZ: Cefazoline; FEP: Cefepime; CTX: Cefotaxime; CTT: Cefotetan: CAZ: Ceftazidime; CRO: Ceftriaxone; CXM: Cefuroxime; GAT: Gatifloxacin; IMP: Imipenem: MEM: Meropenem: LEV: Levofloxacin; F: Nitrofurantoin; TIM: Ticarcillin/Clavulinic; TOB: Tobramycin; SXT: Trimethoprim/sulfamethoxazole; TGC: Tigecycline; CL: Colistin; R: Resistant, S: Sensitive, I: Intermediate ‎ MDR: Multi drug resistant, XDR: Extensive drug resistant.

**Table 5:** Correlation between biocides inhibitory concentration and the presence of qac genes.

Chemical Agent	MIC μg/mL (%)	Number of Isolates (*n* = 22)	qac Genes		Chi-Square Tests	*p*-Value
			Positive	Negative		
Povidone-iodine	>50,000 (5%)	0	0	0	-	-
	<50,000 (5%)	22	15	7		
Chlorhexidine	>75 (0.0075%)	12	11	1	6.712	0.0096 *
	<75 (0.0075%)	10	4	6		
Cetrimide	>750 (0.075%)	17	14	3	6.924	0.0085 *
	<50 (0.075%)	5	1	4		

* The result is significant when *p* < 0.05.

**Table 6:** Correlation between qac genes and *bla*_VIM_ and *bla*_NDM-1_ among MBL-positive *A. baumannii* isolates.

	MBL Genes	*bla*_VIM_		*bla*_NDM-1_	
qac Genes		Positive	Negative	Positive	Negative
Positive		15	0	11	4
Negative		4	3	2	5
*p*-value		0.0064 *		0.0467 *	

* The result is significant when *p* < 0.05.
